# Directional Alignment of Polyfluorene Copolymers at Patterned Solid-Liquid Interfaces

**DOI:** 10.3390/polym9080356

**Published:** 2017-08-11

**Authors:** Xiaolu Pan, Hongwei Li, Xinping Zhang

**Affiliations:** 1 Institute of Information Photonics Technology and College of Applied Sciences, Beijing University of Technology, Beijing 100124, China; xiaolupan@emails.bjut.edu.cn (X.P.); hw0425@126.com (H.L.); 2 Advanced Nano-Materials Division, Suzhou Institute of Nano-Tech and Nano-Bionics (SINANO), Chinese Academy of Sciences (CAS), Suzhou 215123, China

**Keywords:** directional alignment into fibers, polyfluorene copolymers, liquid-solid interfaces, polarization spectroscopy, photoconductivity

## Abstract

Polyfluorene and its derivatives have been recognized as efficient light-emitting semiconductors. However, directional alignment of polyfluorene copolymers at a large scale has rarely been observed, in particular for the two relatively more amorphous members of poly-9,9-dioctylfluorene-co-bethiadisazole (F8BT) and poly-(9,9-dioctylfluorenyl-2,7-diyl)-*co*-(*N*,*N*0-diphenyl)-*N*,*N*′di(p-butyl-oxy-pheyl)-1,4-diamino-benzene) (PFB) molecules. Furthermore, the directional alignment of PFB has not been observed so far due to the triphenylamine units in its molecular structures. We present, in this work, a solution-processible method to achieve large-scale alignment of F8BT and PFB molecules into fibers as long as millimeters in a defined direction. Spin-coating the polymer film on to a glass substrate patterned by one-dimensional dielectric nano-grating structures through interference lithography and subsequent modification using 1,5-pentanediol have been used in all of the preparation procedures. Polymer fibers have been obtained in an arrangement parallel to the grating lines. The microscopic, spectroscopic, and photoconductive performances verified the formation and the quality of these directionally-aligned polymeric fibers.

## 1. Introduction

Orderly arrangement or directional alignment of organic semiconductor molecules is an effective approach to enhance mobility of charge carriers and is, thus, preferred in efficient organic optoelectronic devices [1,2,3,4,5]. Such directional alignment has been extensively observed in small molecules [6,7,8,9,10]. However, large-scale alignment (>100 µm) behavior has rarely been observed in polymers, although it has been demonstrated in a limited number of polymeric semiconductors [11,12,13,14,15]. Polyfluorene is an efficient light-emitting polymer and its derivatives have been used extensively in light-emitting diodes, transistors, and optically-pumped thin-film lasers [16,17,18,19]. Although polyfluorene shows small-scale π-stacking phases in some specially prepared solid films, [20,21,22] and oriented alignment of poly-9,9-dioctylfluorene (PFO), poly-9,9-dioctylfluorene-co-bethiadisazole (F8BT), and Poly-9,9-dioctylfluorenyl-2,7-diyl-co-bithiophene (F8T2) molecules has been studied on rubbed-polyimide-modified substrates [23], large-scale or long-range alignment has not been reported in the films of these polymers.

In this work, we demonstrate large-scale alignment into defined directions of two derivatives of polyfluorene, poly-9,9-dioctylfluorene-co-bethiadisazole (F8BT) and poly-(9,9-dioctyl-fluorenyl-2,7-diyl)-*co*-(*N*,*N*0-diphenyl)-*N*,*N*′di(p-butyl-oxy-pheyl)-1,4-diamino-benzene) (PFB) on a patterned substrate. The direction and the length of the alignment have been controlled by the underneath patterns provided by a one-dimensional dielectric grating. The alignment process was initiated by the surface modification process using an organic solvent of 1,5-pentanediol. Polymer fibers with a length of millimeters, and a diameter ranging from 300 nm to 2 µm, have been produced for both F8BT and PFB, which extended in the same direction as the grating lines. Optical and electrical characterization supply evidence for the directional alignment processes.

## 2. Experimental Methods

### 2.1. Materials and Measurement Methods

The polymers of F8BT and PFB were purchased from Sigma-Aldrich and American Dye Source, Inc. (Quebec, QC, Canada), respectively. 1,5-pentanediol and toluene were obtained from the Sinopharm Chemical Reagent Co., Ltd. (Beijing, China). All of these compounds were used as received. A very commonly used positive photoresist S1805 was purchased from Rohm & Haas (Philadelphia, PA, USA) and filtered through a filtration membrane with a pore size of 200 nm before being used. The atomic force microscope (AFM) images were measured using a WiTec Alpha300S system (WITec Wissenschaftliche Instrumente und Technologie Gmbh, Ulm, Germany). Optical microscopic images were acquired using an Olympus BX51 polarization optical microscope (Olympus Corporation, Tokyo, Japan). The photoluminescence (PL) spectra was taken on a time-correlated single-photon counting system (FLS920) from Edinburg Instruments (Livingston, UK). The absorption spectra were measured using an Agilent G1103A UV-VIS spectrometer (Agilent Technologies, Santa Clara, CA, USA). The polarization dependence of the absorption spectra was controlled by placing a polarizer between the sample and the light source. The electrical performance was characterized by an Agilent 4156C semiconductor parameter analyzer (Agilent Technologies, Santa Clara, CA, USA).

### 2.2. Preparation of the Directionally-Aligned Polymer Fibers

**Figure 1:**
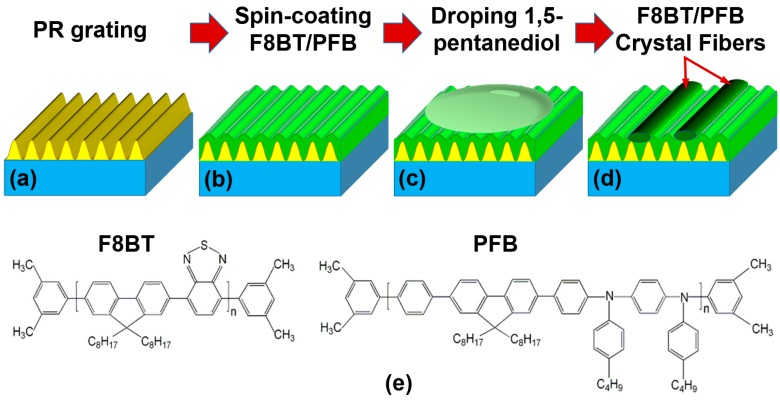
Fabrication procedures for the F8BT fibers. (**a**) The template PR grating with a period of 670 nm on a glass substrate; (**b**) spin-coating of the polymer film; (**c**) drop casting of 1,5-pentanediol onto the top surface of the polymer film; (**d**) growth of the polymeric fibers; and (**e**) chemical structures of F8BT and PFB.

Interference lithography is the first step to produce photoresist (PR) template grating on a silica substrate, as shown in Figure 1a. A 325-nm He-Cd laser (Kimmon Koha Co. Ltd., Tokyo, Japan) was used as the ultraviolet (UV) light source and S-1805 photoresist was used as the photosensitive medium. The PR S1805 is sensitive to UV light at a wavelength shorter than 400 nm and it is hardly dissolved in toluene, but slightly in 1,5-pentanediol, which provided us with a conditional probability to achieve the directional alignment of polymer molecules. The substrate has an area of 15 mm × 15 mm and a thickness of 1 mm. The PR grating employed in this work has a period of about 670 nm. The polymer solution in toluene with a concentration of 15 mg/mL was heated mildly on a hotplate at 60 °C for 20 min before it was spin-coated onto the PR grating at a speed of 2000 rpm for 30 s (Figure 1b). Then, the sample was put into a Petri dish and heated at 80 °C for 20 min on a hotplate, finishing the preparation of the polymer film on the PR grating. After the sample was cooled down in air, 1,5-pentanediol with a volume of about 75 µL was dropped onto the top surface of the sample, as shown in Figure 1c. In the last stage, the sample was annealed at 120 °C for 3.5 h and polymer fibers were grown along the grating lines, as shown in Figure 1d. Similar preparation procedures were adopted for both the F8BT and PFB polymers. The chemical structures of F8BT and PFB are presented in Figure 1e, where PFB exhibits much more amorphous than F8BT.

### 2.3. Device Fabrication and Characterization

Gold (Au) electrodes with a thickness of about 50 nm were deposited by vacuum thermal evaporation through a mask with a channel length of *L* = 2.0 mm and a channel width of *W* = 10 μm at a rate of ca. 0.05 Å per second under a pressure of 10^−4^ Pa. A laser beam at 405 nm with diameter of about 2 mm was used as the excitation in the characterization of the photoconductivity performance.

## 3. Microscopic Characterization

**Figure 2:**
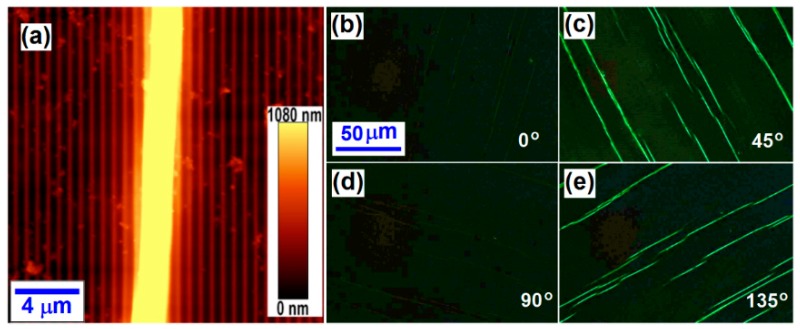
(**a**) AFM image measured on the F8BT fibers prepared on a photoresist grating template; and (**b**–**e**) polarization optical microscopic images of the F8BT fibers.

Figure 2a shows the atomic force microscopic (AFM) image measured on the finished F8BT fibers produced on the template grating structures. From Figure 2a, we cannot only determine the thickness of the directionally-aligned F8BT fibers, but also measure the period of the template grating, which are 2 µm and 676 nm, respectively.

Figure 2b–e show the polarized optical microscopic images of the fibers of the aligned F8BT molecules with the grating lines oriented at 0°, 45°, 90°, and 135°, respectively, with respect to the transmission axis of one of the two orthogonally-arranged polarizers. We can observe bright F8BT fibers only when the grating lines are oriented at 45° and 135° with respect to the transmission axis of the polarizers, which verifies the birefringence or directional alignment properties of F8BT molecules along the direction of the grating lines.

The polymer fibers were produced through the alignment of the F8BT molecules in a direction approximately perpendicular to the fluorene’s rigid plane. Such rigid and planar features of the fluorene structures supplied the basic mechanisms for the directional alignment of polyfluorene and its derivatives. Thus, the direction of the template grating lines actually defines the alignment direction. For the observation under a polarization optical microscope equipped with two orthogonally-oriented polarizers, when the alignment direction is parallel or perpendicular to the transmission axis of either polarizer (the fibers are orientated at 0° or 90° with respect to the polarizers), no light can propagate through the system so that a dark field can be observed under the microscope. However, when the fibers are oriented at 45° or 135° with respect to the transmission axes of the polarizers, maximum transmission of light can be observed. Therefore, this polarization- and orientation-dependent performance of optical transmission supplied effective and reliable evidence for the directional alignment nature of the polyfluorene fibers.

**Figure 3:**
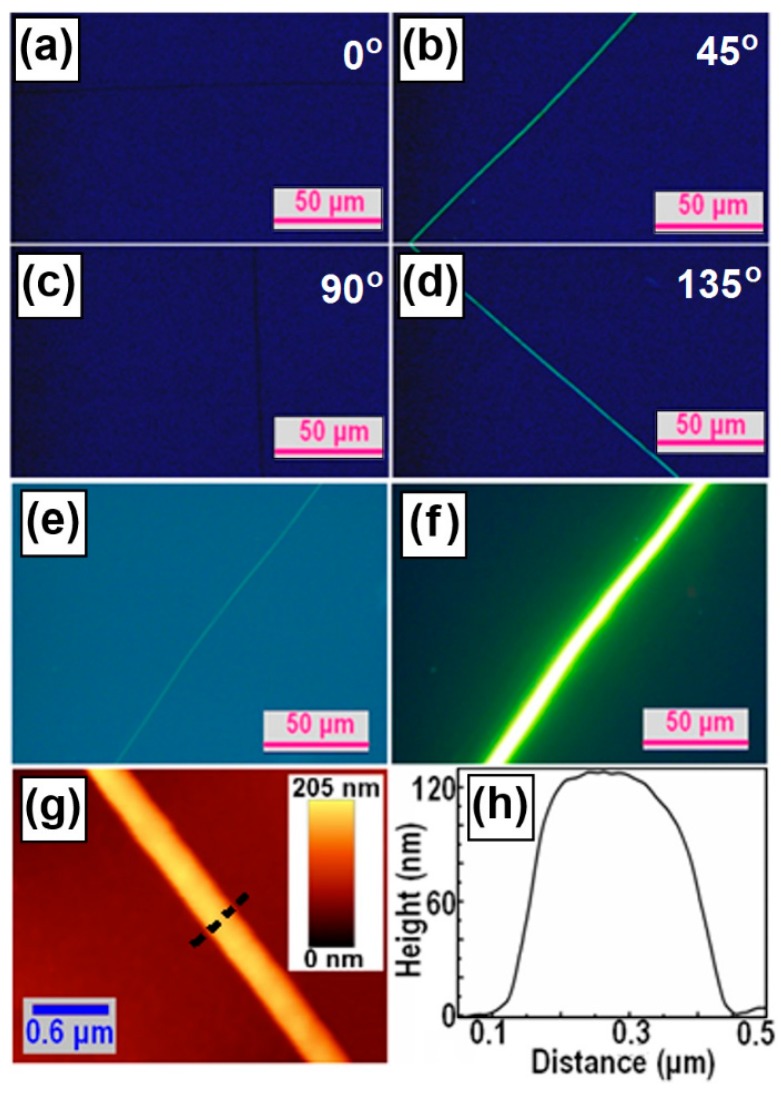
(**a**–**d**) Polarization optical microscopic images of a single F8BT fiber; (**e**,**f**): transmission and fluorescence optical microscopic image of the single F8BT fiber; and (**g**,**h**) AFM height image and plot of the profile of the fiber, respectively.

**Figure 4:**
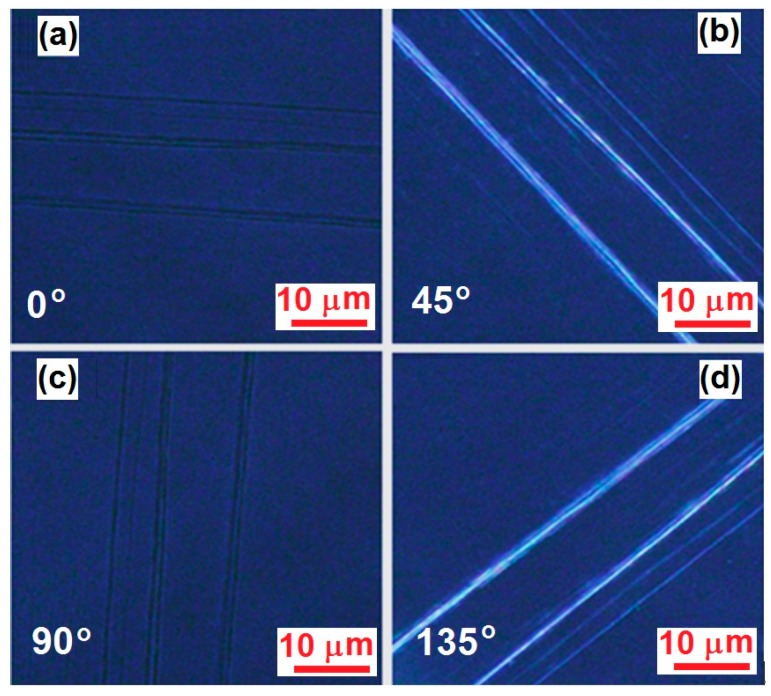
Polarization optical microscopic images measured on the PFB fibers with the grating lines orientated at (**a**) 0°; (**b**) 45°; (**c**) 90°; and (**d**) 135° with respect to one of the two orthogonally-arranged polarizers.

We stress further that although the polymeric fibers were grown on template gratings, they are not distributed periodically on the substrate, which can be observed clearly in Figure 2, as well as in the subsequent Figure 3 and Figure 4. Therefore, the color and transmission change with polarization through the fibers in these measurement data definitely did not result from the diffraction processes.

The directional alignment of F8BT molecules the PR grating substrate has been based on the following mechanisms: (1) Slight dissolution of photoresist in 1,5-pentanediol enabled swelling of the PR grating lines and narrowing of the channel width between two adjacent grating lines; (2) The F8BT molecules detached from the solid film and dissolved into 1,5-pentanediol gradually, producing a layer of saturated solution on the tops of the grating-patterned substrate; (3) With evaporation of 1,5-pentanediol, the F8BT molecules solidified out and assembled in the narrowed channels into seeds through π-π stacking, which are the basis for further alignment; (4) Long-range alignment of the F8BT molecules from the seeds along the grating grooves.

However, the F8BT fibers shown in Figure 2 are aligned along the direction of the grating lines and have random distributions in their length and separation distance. The quality of the F8BT fibers can be improved by modifying the fabrication process, where continuous single fibers may be selectively achieved by removing the template grating and the low-quality fibers. A series of test experiments enabled optimization of the fabrication parameters, which involve a F8BT/toluene solution concentration of 6 mg/mL, a spin-coating speed of 2200 rpm, an annealing temperature of 120 °C, and an annealing solvent of 1,5-pentanediol. The resultant sample was then immersed in acetone for 20 min to remove the template PR grating. Due to a continuous F8BT film underneath the fibers and above the PR grating, the fibers were not removed as the photoresist was dissolved into acetone. In the last stage, the sample was processed by low-power ultrasonic for about 10 s to remove the F8BT thin film and low-quality short fibers.

Figure 3a–d present the optical polarization microscope images of a single F8BT fiber at an orientation angle of 0°, 45°, 90°, and 135° with respect to the transmission axis of one of the polarizers. Clearly, the bright images of the F8BT fiber with dark backgrounds can be observed only at an orientation angle of 45° and 135°, verifying the directional alignment performance of the F8BT fibers. Figure 3e shows the transmission optical microscope image without polarization performance and Figure 3f shows the fluorescence optical microscope image under UV-light excitation. Strong photoluminescence can be observed from the single F8BT fiber in Figure 3f. It can thus be confirmed from Figure 3e,f that both the template PR grating and the continuous F8BT thin film have been removed completely. Figure 3g,h show the AFM height image and the profile plot of the single F8BT fiber, which has a width of about 350 nm and a height of about 120 nm.

According to Figure 1e, PFB molecules are much more amorphous than F8BT. However, similar fabrication procedures also applied well to PFB and the directional alignment was achieved on the pattern substrate with the aid of the annealing solvent. Figure 4a–d show the polarization optical microscopic images measured on the fibers of directionally-aligned PFB molecules, corresponding to the orientation of the grating lines at 0°, 45°, 90°, and 135°, respectively, with respect to one of the two orthogonally mounted polarizers. Clearly, the preparation method for F8BT fibers also applies to PFB. Although the thickness, length, and continuity of the PFB fibers are randomly distributed at different locations, the fibers are aligned very well in the direction of the grating lines.

## 4. Spectroscopic Characterization

**Figure 5:**
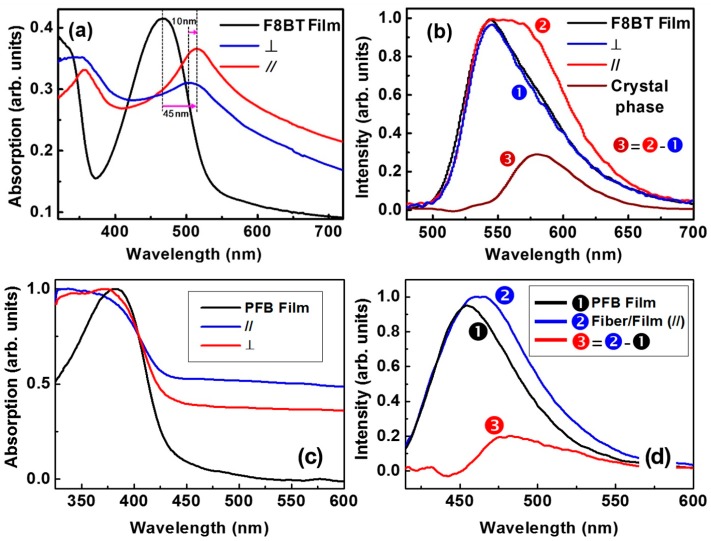
(**a**) Absorption spectra of the spin-coated F8BT film (black) and the F8BT fibers for light polarized perpendicular (blue) and parallel (red) to the grating lines; (**b**) The corresponding PL spectra for the sample in (**a**); (**c**) Absorption spectra of the spin-coated PFB film (black) and the PFB fibers for light polarized perpendicular (red) and parallel (blue) to the grating lines; and (**d**) The PL spectra measured on the PFB film (black), PFB fibers at a parallel polarization direction (blue), and the difference between them (red).

Figure 5a shows the absorption spectra of the spin-coated F8BT film (black) and the F8BT fibers in Figure 3 aligned along the PR gratings with the light polarized perpendicular (blue) and parallel (red) to the grating lines. The fibers show redshifted absorption from 468 to 513 nm with respect to the continuous film for a parallel polarization. This is commonly observed for π-stacking of organic molecules due to intermolecular coupling along the stacking axis [24,25,26]. There is also a redshift of about 10 nm between the blue and red spectra, however, this redshift is much smaller than that relative to the spin-coated film, which implies inclination of the molecule plane with respect to the stacking axis and some disorders during the stacking process [27,28,29,30].

Figure 5b shows the PL spectra of the spin-coated F8BT film (black) and the fibers for polarization perpendicular (blue) and parallel (red) to the fibers. An obvious broadening of the PL spectrum can be observed for a parallel polarization, implying new structures were produced in the excited states of the fiber that inducing new features in the emission spectrum.

Curve ➌ in Figure 5b is a difference spectrum between the red (➋) and the blue (➊) ones, which is resolved as the emission from the π-stacking phase in the F8BT fibers and peaks at about 580 nm. Therefore, the face-to-face intermolecular coupling between π-stacked F8BT molecules induced a lower-leveled band of the excited states, which is not observable in spin-coated films. Thus, the spectroscopic performances in Figure 5 supplies more solid evidence for the directional alignment of the F8BT molecules.

Figure 5c,d show similar measurements on the PFB samples described in Figure 4. Figure 5c shows the absorption spectra measured on a solid film (black), on the fibers at a parallel (red) and perpendicular (blue) polarization direction. It should be noted that PFB has an absorption mainly in the UV and blue spectral range, which overlaps the absorption by the photoresist. Therefore, although the PFB fibers show obvious red-shifted absorption spectra with respect to the films, in particular for the polarization direction parallel to the fibers, the absorption spectra have been modulated by the substrate absorption. In contrast, the PL spectra are more reliable for the characterization of the spectroscopic properties of the PFB fibers. As shown in Figure 5d, the PFB fibers show a broadened and redshifted PL spectrum for a parallel (➋) polarization with respect to the film (➊). If we assume that the fibers have a similar PL spectrum as the film in the perpendicular polarization, the difference between spectra ➋ and ➊ shows a rough evaluation on the PL spectrum of the π-stacking phase, as shown by spectrum ➌, which peaks at about 480 nm and extends in the blue and green.

Thus, both the optical microscopic characterization in Figure 2, Figure 3 and Figure 4 and the spectroscopic characterization in Figure 5, which show clear birefringence with strong polarization dependence, have consistently verified the directional alignment nature of the F8BT and PFB molecules in fibers grown along the template grating grooves.

## 5. Photoconductivity Characterization

**Figure 6:**
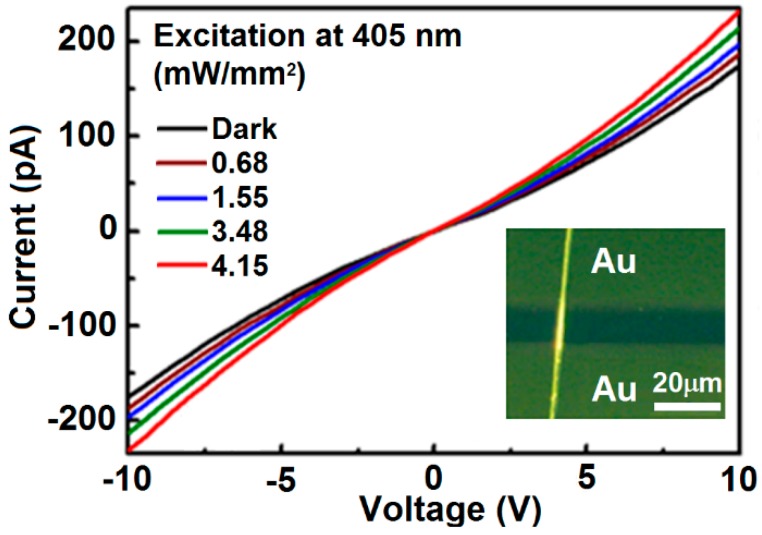
I–V curves measured on the photoconductive device based on a single F8BT fiber under different excitation power density. Inset: optical microscopic image of the device.

Figure 6 shows the I-V curves measured on photoconductive device based on a single F8BT fiber in Figure 3 under different excitation power density. The inset of Figure 6 shows the optical microscopic image of the device. Gold was deposited onto the F8BT fiber and functioned as electrodes, leaving a channel as wide as 10 µm. At an applied voltage of 10 V, the dark current of the device is about 170 pA. With the increase of the excitation intensity at 405 nm, the photocurrent of the device was increased from 170 to 230 pA. The performance in Figure 6 implies excellent continuity of the F8BT fiber with good conductivity. However, a slow increase in the photocurrent with increasing the laser power density at 405 nm was observed in Figure 6, this is mainly due to the low absorption by the F8BT fibers at 405 nm, as can be seen in Figure 5a, and the small amount of the fibers with low density within the studied area.

## 6. Conclusions

Directional alignment of F8BT and PFB molecules into large-scale fibers has been achieved by the solvent-annealing in 1,5-pentanediol of the spin-coated F8BT and PFB films on the photoresist grating structures. Microscopic and polarization-dependent spectroscopic characterization verified convincingly the directional π-stacking performance of the F8BT and PFB fibers. Measurements on the photoconductive performance show excellent continuity and conductivity of the single F8BT fibers. This is a first demonstration of millimeter-to-centimeter-scale directional alignment of the polyfluorene molecules, which can be applied in efficient light-emitting or transistor devices based on polyfluorene copolymers. Furthermore, the preparation method for polymeric fibers in this work may be explored to become applicable to other polymeric semiconductors.
